# Mental Health and Well-Being of Undergraduate Nursing Students: A Cross-Sectional Study Using Canonical Correlation Analysis

**DOI:** 10.3390/ejihpe15090169

**Published:** 2025-08-23

**Authors:** Luís Loureiro, Amorim Rosa, Tânia Morgado, Rosa Simões

**Affiliations:** Health Sciences Research Unit: Nursing (UICISA: E), Nursing School of Coimbra, 3046-851 Coimbra, Portugal; amorim@esenfc.pt (A.R.); tmorgado@esenfc.pt (T.M.); rosasimoes@esenfc.pt (R.S.)

**Keywords:** mental health, well-being, nursing, higher education

## Abstract

**Background:** In recent decades, the relationship between mental health and well-being has been explored from many perspectives, with emphasis on the two-continua model of health in different contexts, with an emphasis on young higher education students. Both mental health and well-being are considered predictors of academic success. This study aims to analyze the relationship between mental health and well-being among first- and fourth-year nursing students. The sample consisted of 473 nursing students from a university in the central region of mainland Portugal. **Methods:** Data were collected using the short versions of the Depression, Anxiety and Stress Scale (DASS-21) and the Mental Health Continuum—Short Form (MHC-SF). **Results:** Canonical correlation analysis revealed a significant negative association between psychological distress and mental well-being. The first statistically significant canonical function (*p* < 0.05; Cr = 0.601) was primarily defined by depression (canonical loading = −0.992) in the distress group and emotional well-being (canonical loading = 0.948) in the well-being group. Redundancy analysis confirmed a significant interdependence: variables related to psychological distress explained 27.8% of the variance in well-being, while well-being variables explained 23.8% of the variance in distress. **Conclusions:** These results reinforce the two-continua model, highlighting the need to address both mental health and well-being throughout higher education.

## 1. Introduction

In recent decades, a robust body of evidence has shown that mental health (MH) and well-being (WB), although distinct concepts, are closely interconnected (Keyes, 2002, 2005) and play a critical role in the academic success of higher education (HE) students (Cuppen et al., 2024; Hernández-Torrano et al., 2020). This evidence is associated with the development of the two-continua model of MH proposed by Keyes (2002, 2005), which conceptualizes MH not only as the absence of psychopathology but also as the presence of positive emotional, psychological, and social functioning. According to this model, individuals with low levels of distress can experience low WB, while others, despite exhibiting psychopathological symptoms, can flourish (Westerhof & Keyes, 2009; L. Loureiro, 2024).

Evidence suggests that HE students are particularly vulnerable to mental health (MH) issues associated with low WB, especially those enrolled in health-related programs, particularly nursing (Patelarou et al., 2021; Purnama et al., 2021; Sonmez et al., 2023; Efstathiou et al., 2025).

In the case of nursing students, evidence suggests that their increased psychological vulnerability is linked to factors such as intense academic demands, the emotional challenges associated with healthcare provision, and early exposure to human suffering during clinical placements (Patelarou et al., 2021; Patterson et al., 2025). WB is increasingly recognized as a key factor in HE students’ academic performance, influencing their motivation, concentration, and persistence throughout their studies (Candeias et al., 2019).

In HE, two moments are particularly significant in students’ academic journeys. The first occurs during the transition to HE, a critical period involving a series of changes and adaptation requirements that can negatively impact students’ MH and WB (Pointon-Haas et al., 2023; Osafo et al., 2025). This impact may be even greater for students with a history of emotional difficulties or mental disorders (Auerbach et al., 2016; Peris-Baquero et al., 2023; Zhang et al., 2024). The second critical moment occurs at the end of the nursing program, particularly in the fourth year, when students face the transition to the job market and the expectation of professional integration, as well as dissatisfaction with the program (Sonmez et al., 2023). This period can generate feelings of uncertainty, anxiety, and emotional overload, which negatively influence emotional, psychological, and social WB. Some studies even suggest a progressive decline in nursing students’ MH throughout their training (Sonmez et al., 2023).

Despite evidence of increased psychological distress among nursing students, some studies suggest that certain factors associated with nursing training can protect MH. These factors include the development of a professional identity, a sense of belonging to an academic community, and the perception of the profession’s social value, which are associated with greater psychological resilience and satisfaction with the program (Galvin et al., 2015; Jafarianamiri et al., 2022). In addition, participation in clinical placements contributes to the development of socio-emotional skills, empathy, and self-efficacy, which in turn improve emotional adjustment and WB (Moeini et al., 2019).

Therefore, it is possible that, in certain contexts, the nursing program contributes to students’ personal and emotional development, mitigating the negative effects of academic stress.

The recent literature advocates for an integrative approach that simultaneously assesses MH and WB indicators (Hernández-Torrano et al., 2020), providing a more comprehensive understanding of the factors that influence nursing students’ psychological adjustment. Several factors have been associated with MH in this population. Social support (from peers, teachers, and family) consistently emerges as a protective factor (Massano-Cardoso et al., 2024). Mental health literacy, defined as the knowledge and beliefs about mental disorders that facilitate their recognition, management, and prevention (Jorm, 2015), has been positively associated with higher levels of WB and more positive attitudes toward help-seeking (Tambling & D’Aniello, 2023; L. M. J. Loureiro et al., 2025). In addition, effective coping strategies, such as planning, positive reappraisal, and emotional support, contribute to emotional regulation and reduce the impact of academic stress (Rafati et al., 2017).

However, despite the growing attention to the MH of HE students, there is a gap in the literature regarding the integrated analysis of the association between negative (stress, anxiety, and depression) and positive indicators (WB dimensions) of MH. Few studies have used canonical correlation analysis (CCA) to explore the simultaneous and complex relationship between these two domains.

This study aims to analyze the association between levels of stress, anxiety, and depression (as assessed by the Depression, Anxiety and Stress Scale—DASS-21) and the dimensions of mental WB (as assessed by the Mental Health Continuum—Short Form—MHC-SF) in a sample of nursing students from a university in the central region of mainland Portugal.

## 2. Materials and Methods

### 2.1. Study Design

This study follows a quantitative approach with a correlational, cross-sectional design.

### 2.2. Participants

The sample consisted of 473 nursing students from a university in the central region of mainland Portugal, including 281 first-year students and 192 fourth-year students. In terms of gender, 81 students were male and 392 were female. The mean age was 20.41 years (*SD* = 4.72 years), with first-year students averaging 19.25 years (*SD* = 5.24) and fourth-year students averaging 22.11 years (*SD* = 3.14).

The *t*-test revealed significant differences in age by course year (*t*(471) = −6.781; *p* < 0.001; *d* = 0.64), reflecting the expected academic progression, with fourth-year students being older than their younger colleagues.

### 2.3. Instruments

The first section of the questionnaire includes sociodemographic questions about gender, age, course year, and whether students had relocated their residence to attend the program.

#### 2.3.1. Depression, Anxiety and Stress Scale (DASS-21)

This study used the DASS-21, developed by Lovibond and Lovibond (1995) and translated into Portuguese by Pais-Ribeiro et al. (2004). This version consists of 21 items, with seven items corresponding to each of the three subscales, namely depression, anxiety, and stress. Participants are asked to rate the extent to which they experienced each symptom over the past week on a 4-point Likert scale, ranging from *Did not apply to me at all* (0) to *Applied to me very much, or most of the time* (3).

In this study, the DASS-21 showed good reliability indices, with *α* = 0.89 for depression, *α* = 0.86 for anxiety, and *α* = 0.89 for stress.

#### 2.3.2. Mental Health Continuum—Short Form (MHC-SF)

To assess mental WB, this study used the MHC-SF developed by Keyes et al. (2008) in the version translated and adapted for the Portuguese population by Matos et al. (2010). This 14-item instrument uses a Likert-type response scale ranging from 0 (*never*) to 5 (*every day*). The MHC-SF assesses three dimensions of WB, including emotional well-being (EWB), social well-being (SWB), and psychological well-being (PWB). Based on the answers to the items, the MHC-SF classifies individuals into one of three MH states, which are flourishing, moderate, or languishing. Accordingly, those who report high well-being on at least one of three emotional well-being measures and high well-being on any six of the eleven psychological and social well-being measures are defined as flourishing. In contrast, those who report low levels of well-being on at least one of the emotional measures and low well-being on at least six of the psychological and social well-being measures are defined as languishing. The rest who do not fulfill the criteria of flourishing or languishing are defined as moderately mentally healthy.

In this study, the MHC-SF showed high reliability, with *α* = 0.87 for EWB (3 items), *α* = 0.86 for SWB (5 items), and *α* = 0.90 for PWB (6 items).

### 2.4. Data Collection

Data were collected at a university in the central region of mainland Portugal between September and October of 2024. All first- and fourth-year nursing students were invited to participate in the study. Data were gathered in classrooms using the Google Forms platform, with the researchers present. Participants accessed the questionnaires via a QR code and provided electronic informed consent, confirming their understanding of the study’s objectives and their voluntary participation.

### 2.5. Statistical Analysis

Data were analyzed using SPSS Version 29.

Appropriate summary statistics were calculated, as well as absolute and percentage frequencies, where the type of variable justified it. The normal distribution of the variable in the population was analyzed using the Lilliefors-corrected Kolmogorov–Smirnov test. Levene’s test was used to assess the homogeneity of variance.

The following bivariate analyses were performed: the *t*-test for independent groups (using Cohen’s *d* to measure effect size) and the Pearson’s correlation coefficient significance test. Effect size can be defined as the degree to which a phenomenon is present in the population (Cohen, 1988).

Reliability was assessed based on the internal consistency of each subscale of the MHC-SF and DASS-21, using Cronbach’s alpha coefficients.

To address the study’s main objective, CCA was used. This multivariate statistical technique is used to analyze the relationship between two sets of variables by identifying the linear combinations that maximize the correlation between them. The decision to employ canonical correlation analysis (CCA) was made for a variety of reasons. Primarily, the study’s exploratory approach aligns well with the multivariate characteristics of the variables being studied, despite its correlational nature. CCA is adept at revealing intricate patterns within numerous correlations and effectively capturing the extent to which the two sets of variables are interrelated. An important benefit of CCA is its ability to examine variables without imposing strict causal assumptions, a critical aspect given the reciprocal influence that mental health and well-being factors can have on each other.

In this study, it was used to assess the association between the three MH dimensions of the DASS-21 and the three factors of the MHC-SF. The aim is to understand how the underlying dimensions in one set of variables relate to the underlying dimensions in the other set. In this study, the MH variables are represented by the letter *M* and the WB variables by the letter *W*.

### 2.6. Ethical Approval

This study is part of a project registered with the Health Sciences Research Unit: Nursing (UICISA: E), entitled *Mental health literacy and first aid: Preventive mental health and mental health promotion*. The questionnaire was approved by the Nursing School of the University of Coimbra and the UICISA: E Ethics Committee (no. P603-06/2019). The following inclusion criteria were applied: agreeing to participate voluntarily in this study and signing the electronic consent form.

## 3. Results

### Mental Health and Well-Being (By Course Year)

Before performing the CCA, the mean scores of the DASS-21 and MHC-SF subscales were compared by course year. As shown in Table 1, statistically significant differences were found only in anxiety (*t*(473) = 3.560, *p* < 0.001; *d* = 0.33), indicating a medium effect size. No statistically significant differences were found in the other subscales.

Bivariate correlation coefficients were calculated to analyze the associations between MH and WB variables. As shown in Table 2, all correlations were negative, linear, and statistically significant, ranging from moderate (*r* = −0.311; *p* < 0.01) to substantial (*r* = −0.566; *p* < 0.01).

The strongest correlations were found between the WB subscales and the depression subscale (−0.482 < *r* < −0.556), followed by the correlations between the stress subscale and the WB subscales (−0.424 < *r* < −0.384). The weakest correlations were found between anxiety and WB (−0.311 < *r* < −0.350). These findings indicate a trend: higher levels of WB are associated with fewer symptoms of mental illness and vice versa.

CCA was then conducted, grouping the three DASS-21 subscales into one variable set (M) and the three MHC-SF subscales into the other set (W). As shown in Table 3, the analysis identified three canonical functions; however, only the first two were statistically significant.

The first canonical function revealed a substantial correlation (*r* = 0.601), with an associated eigenvalue of 0.567 (proportion of variance explained by the canonical function). Wilks’ lambda for this model was statistically significant (Λ = 0.623; *F*_(9; 1136)_ = 27.143; *p* < 0.001), showing a significant association between the two sets of variables (M and W).

The second canonical function had a low correlation (*r* = 0.155), with an associated eigenvalue of 0.025 (proportion of variance explained by the canonical function). The Wilks’ lambda for this model was statistically significant (Λ = 0.976; *F*_(4; 936)_ = 2.909; *p* < 0.05), confirming a significant association between the two sets of variables (M and W).

The third canonical function was not statistically significant and therefore did not contribute to the association between the two sets of variables.

Table 4 shows the canonical loadings, which represent the correlations between the original variables and their respective canonical variables.

For the first set of variables related to MH (M), the canonical correlation was 0.601. This function captures the strongest relationship between the two sets of variables. Three negative correlations were found, namely very strong for depression (−0.992), strong for stress (−0.768), and moderate for anxiety (−0.616). These results suggest that the first canonical variable from set 1 (M) is primarily defined by depression, followed by stress and anxiety.

For the mental WB set (W), the first canonical function revealed three positive correlations, which were very strong for EWB (0.948) and strong for PWB (0.859) and SWB (0.808). The first canonical variable from set 2 (W) was primarily defined by EWB, PWB, and SWB.

Overall, the first canonical function was characterized by a strong inverse relationship between psychological distress and WB. Students with higher levels of psychological distress, mainly driven by depressive symptoms, tend to have lower mental WB scores, with EWB emerging as the most significant component.

The second canonical function was less important and had a lower value. Although statistically significant, it captured a different dimension from the first function. In the mental health (M) set, there was a strong negative loading for stress (–0.663) and a moderate negative loading for anxiety (–0.590), indicating a stress–anxiety dimension. In the W set, SWB was the variable with significance, showing moderate-to-strong negative loadings. The contributions of EWB (−0.120) and PWB (−0.120) were residual. These results suggest that the second canonical function reflects an inverse association between stress and anxiety and SWB: an increase in stress and anxiety is associated with a decrease in social well-being.

The analysis of the cross-loadings (Table 4) indicates that, in the first canonical function, depression from the M set and EWB from the W set are the strongest predictors of the canonical variable in the opposite set. This finding suggests that depressive symptoms are a key indicator of overall WB, while EWB is an indicator of psychological distress. The cross-loadings for the second canonical function were very low, suggesting that the M and W sets are poor predictors of the canonical variables in the opposite set.

Table 5 shows the proportion of variance explained by the canonical functions within each set of variables (M by M or W by W) and between the sets of variables (redundancy).

For the first canonical function, the canonical variable explained 65.2% of the total variance in set M (psychological distress variables) and 76.3% of the total variance in set W (WB variables). These findings suggest that the canonical variables effectively capture the variability within their respective sets of original variables. With regard to redundancy, the first canonical function revealed that the canonical variable from set W explained 23.8% of the variance in set M, while the canonical variable from set M explained 27.8% of the variance in set W.

Although the second canonical function was statistically significant, its explanatory power was very limited.

## 4. Discussion

The main aim of this study was to analyze the association between MH (as assessed by the DASS-21) and WB (as assessed by the MHC-SF) in a sample of nursing students using CCA. It aimed to assess differences in students’ MH and WB by course year.

The results showed that first-year students reported significantly higher levels of anxiety (*p* < 0.001) and stress (*p* < 0.05), although effect sizes were small. These findings are consistent with evidence indicating that first-year nursing students tend to experience higher levels of stress and anxiety than fourth-year students (Visier-Alfonso et al., 2024). This increase may be due to the fact that the transition into university is a critical period characterized by demands for emotional, academic, and social adaptation (Candeias et al., 2019; Buote et al., 2007).Thus, first-year students are more likely to report higher levels of anxiety and stress, particularly those with a history of emotional difficulties (Peris-Baquero et al., 2023).

Course attendance may also play a key role in how students develop resilience and learn to manage stress and anxiety on a daily basis. These results may be partly due to fourth-year students having a higher level of mental health literacy than first-year students (L. Loureiro et al., 2025), which enables them to manage everyday challenges more effectively.

With regard to CCA, the first canonical function revealed a significant correlation (Cr = 0.601), indicating a negative and statistically significant association between the two sets of variables. This finding corroborates Keyes’s (2005) two-continua model of MH, which posits that psychological distress and well-being are distinct but interrelated dimensions (L. Loureiro, 2024). Thus, these results support the idea that the presence of WB is as essential to MH as the absence of mental illness.

The first canonical function was characterized by strong negative contributions from depression (−0.992), stress (−0.768), and anxiety (−0.616) and positive contributions from EWB (0.948), PWB (0.859), and SWB (0.808). The cross-loadings reinforce this pattern, with depression and EWB emerging as the main axes of this association. These results align with studies that analyze depression from the perspective of dynamic systems, such as those by Cramer et al. (2016), which point to complex interactions between symptoms that are responsible for its maintenance and recurrence.

The negative and significant correlation between depression and EWB (*r* = −0.566) highlights the impact of depressive symptoms on students’ emotional balance. This finding is consistent with studies identifying depression as a relevant predictor of reduced EW (Liu & Wang, 2024). Similarly, the negative correlations between stress and anxiety and the psychological and social dimensions of WB reinforce the role of these symptoms as barriers to adaptive functioning in the academic environment (Shah et al., 2022).

The redundancy analysis revealed that the psychological distress variables explained 27.8% of the variance in WB, while WB variables explained 23.8% of the variance in the psychological distress. These results reveal a relevant interdependence between the two domains and confirm the value of CCA as a useful tool for researching MH in an academic context.

The current literature advocates for an integrative approach to MH that goes beyond the absence of disease and promotes flourishing, which is defined as a state of optimal emotional, psychological, and social functioning (Keyes, 2014). Although this study did not directly assess flourishing, languishing, or moderate states, future research should explore these profiles, given their usefulness in identifying groups with different support needs.

Although the second canonical function was statistically significant, it showed a weak correlation (*r* = 0.155) and low explanatory power. It points to a possible association between stress and anxiety levels and lower SWB, suggesting that students who are more anxious or overburdened may experience a reduced sense of belonging and social support. This phenomenon has already been described in the literature on social withdrawal in university settings (Galvin et al., 2015).

It should also be noted that the predominance of women in the sample (82.8%) and the use of a written self-report may have influenced the reported levels of psychological distress, as women are generally more likely to express emotional symptoms (Chaplin & Aldao, 2013). The literature also suggests that sociocultural factors and more internalizing coping styles contribute to women’s greater emotional vulnerability. Therefore, MH intervention programs should use gender-sensitive approaches adapted to different styles of emotional expression.

The significant age differences across the years of the program (*p* < 0.001) also highlight the importance of adjusting interventions to the specific needs of each academic phase. Younger students face challenges related to adaptation and vocational uncertainty, while final-year students experience anxiety associated with entering the job market. Phased interventions that integrate early prevention, promote resilience, and support a professional transition could prove particularly effective.

Despite the relevance of these results, this study has some limitations. First, the cross-sectional design and the nature of statistical analyses limit the ability to draw causal inferences. Second, the sample was drawn from a single institution and included “extreme” groups, namely first- and fourth-year students. Third, the fact that other sociodemographic and clinical variables such as socioeconomic status, family status, and distance from family, as well as familiarity with mental illness, were not analyzed could have provided an additional contribution to the interpretation and analysis of the results.

Future studies should include students from all academic years to identify profiles, adopt longitudinal methodologies, or integrate contextual variables, such as social support, family status, distance from family, emotional literacy, socioeconomic status, and academic involvement. The absence of these variables is a major limitation of this study.

Nevertheless, this study makes a significant contribution to the integrated understanding of MH and WB among nursing students. It also serves as an important sub-study for validating the two-continua model of MH. Promoting mental health literacy, strengthening emotional self-regulation, and creating inclusive academic environments are strategies that could have a significant impact on this population.

## 5. Conclusions

The results of this study provide empirical support for Keyes’ two-continua model of MH, highlighting the strong interdependence between the presence of symptoms and low levels of WB. The predominance of depression in the psychological distress dimensions and of EWB in the WB dimensions, as the main drivers of this relationship, underlines the most critical aspects of MH in this population of nursing students.

The higher levels of stress and anxiety among first-year students confirm their vulnerability during the initial stage of their academic journey. They also reinforce the need to develop intervention strategies that integrate MH and WB and are adjusted to each academic stage.

Regarding well-being, the groups of first- and fourth-year students do not differ. These data suggest the need for interventions throughout the training cycle. Strategies such as positive MHL programs, focused on managing MH and WB in daily life, as well as MH first aid programs, could increase WB and reduce psychological distress among nursing students. Integrating MH-promoting programs can also improve nursing students’ MH and WB.

## Figures and Tables

**Table 1:** Mental health and well-being by course year.

Mental Health	Year	n	Mean	*SD*	*t*	*ES* ^(a)^
Stress	1st	281	14.23	9.61	1.961 *	0.18
	4th	192	12.50	9.21		
Anxiety	1st	281	10.06	9.08	3.560 ***	0.33
	4th	192	7.16	8.17		
Depression	1st	281	8.43	7.95	1.770 ^ns^	0.16
	4th	192	7.10	8.13		
**Mental well-being**						
EWB	1st	192	3.70	0.82	0.938 ^ns^	0.09
	4th	281	3.62	0.87		
SWB	1st	192	2.60	1.11	0.204 ^ns^	0.02
	4th	281	2.62	1.00		
PWB	1st	192	3.40	1.03	0.781 ^ns^	0.26
	4th	281	3.47	0.97		

* *p* < 0.05; *** *p* < 0.001; ns: non-significant; ^(a)^ *effect size*: Cohen’s *d* measure.

**Table 2:** Pearson’s correlation matrix of mental well-being dimensions and mental health symptoms (N = 473).

	Stress	Anxiety	Depression
EWB	−0.424 **	−0.335 **	−0.566 **
SWB	−0.433 **	−0.350 **	−0.482 **
PWB	−0.384 **	−0.311 **	−0.514 **

** *p* < 0.01.

**Table 3:** Canonical functions, parameters, and significance tests (*N* = 473).

Function	*r*	Eigenvalue	Λ	*F*	Num D.F	Denom D.F.
1	0.601	0.567	0.623	27.143 ***	9	1136
2	0.155	0.025	0.976	2.909 *	4	936
3	0.016	0.000	1.000	0.120	1	469

*** *p* < 0.001; * *p* < 0.05; Λ = Wilk’s statistic.

**Table 4:** Canonical loadings and cross-loadings (Cl values in parentheses).

Set M	Canonical Loadings		
	1	2	3
Stress	−0.768 (−0.464)	−0.633 (−0.091)	−0.093 (−0.001)
Anxiety	−0.616 (−0.372)	−0.590 (−0.085)	0.522 (0.008)
Depression	−0.992 (−0.599)	0.053 (0.008)	0.111 (0.002)
**Set W**	**1**	**3**	**3**
EWB	0.948 (0.572)	−0.141 (−0.020)	0.285 (0.004)
SWB	0.808 (0.488)	0.579 (0.083)	−0.112 (−0.002)
PWB	0.859 (0.519)	−0.120 (−0.017)	−0.498 (−0.007)

Cl = cross-loadings.

**Table 5:** Proportion of explained variance.

Canonical Variable	M by M	M by W	W 2 by W	W by Set M
1	0.652	0.238	0.763	0.278
2	0.251	0.005	0.123	0.003
3	0.098	0.001	0.114	0.000

## Data Availability

The data presented in the paper are available upon request from the corresponding author.

## Abbreviations

The following abbreviations are used in this manuscript:

CCA	Canonical correlation analysis
CF	Canonical function
DASS	Depression, Anxiety and Stress Scale
DF	Degree of freedom
EWB	Emotional well-being
HE	Higher education
M	Mean
MH	Mental health
MHC	Mental health continuum
PWB	Psychological well-being
SD	Standard deviation
SWB	Social well-being
WB	Well-being

## References

[B1-ejihpe-15-00169] Auerbach R. P., Alonso J., Axinn W. G., Cuijpers P., Ebert D. D., Green J. G., Hwang I., Kessler R. C., Liu H., Mortier P., Nock M. K., Pinder-Amaker S., Sampson N. A., Aguilar-Gaxiola S., Al-Hamzawi A., Andrade L. H., Benjet C., Caldas-de-Almeida J. M., Demyttenaere K., Bruffaerts R. (2016). Mental disorders among college students in the World Health Organization World Mental Health Surveys. Psychological Medicine.

[B2-ejihpe-15-00169] Buote V. M., Pancer S. M., Pratt M. W., Adams G., Birnie-Lefcovitch S., Polivy J., Wintre M. G. (2007). The importance of friends: Friendship and adjustment among 1st-year university students. Journal of Adolescent Research.

[B3-ejihpe-15-00169] Candeias A. A., Alves A. R., Assis C., Fernandes C., Dias C., Pereira R., Candeias A. A. (2019). Bem-estar e vulnerabilidade ao stresse em estudantes do ensino superior. Desenvolvimento ao longo da vida: Aprendizagem, Bem-estar e Inclusão.

[B4-ejihpe-15-00169] Chaplin T. M., Aldao A. (2013). Gender differences in emotion expression in children: A meta-analytic review. Psychological Bulletin.

[B5-ejihpe-15-00169] Cohen J. (1988). Statistical power analysis for the behavioral sciences.

[B6-ejihpe-15-00169] Cramer A. O., van Borkulo C. D., Giltay E. J., van der Maas H. L., Kendler K. S., Scheffer M., Borsboom D. (2016). Major depression as a complex dynamic system. PLoS ONE.

[B7-ejihpe-15-00169] Cuppen J., Muja A., Geurts R. (2024). Well-being and mental health among students in European higher education (EU-ROSTUDENT 8 topical module report).

[B8-ejihpe-15-00169] Efstathiou M., Kakaidi V., Tsitsas G., Mantzoukas S., Gouva M., Dragioti E. (2025). The prevalence of mental health issues among nursing students: An umbrella review synthesis of meta-analytic evidence. International Journal of Nursing Studies.

[B9-ejihpe-15-00169] Galvin J., Suominen E., Morgan C., O’Connell E. J., Smith A. P. (2015). Mental health nursing students’ experiences of stress during training: A thematic analysis of qualitative interviews. Journal of Psychiatric and Mental Health Nursing.

[B10-ejihpe-15-00169] Hernández-Torrano D., Ibrayeva L., Sparks J., Lim N., Clementi A., Almukhambetova A., Nurtayev Y., Muratkyzy A. (2020). Mental health and well-being of university students: A bibliometric mapping of the literature. Frontiers in Psychology.

[B11-ejihpe-15-00169] Jafarianamiri S. R., Qanbari Qalehsari M., Zabihi A. (2022). Investigating the professional identity and resilience in nursing students during the COVID-19 pandemic. Journal of Education and Health Promotion.

[B12-ejihpe-15-00169] Jorm A. F. (2015). Why we need the concept of “mental health literacy”. Health Communication.

[B13-ejihpe-15-00169] Keyes C. L. M. (2002). The mental health continuum: From languishing to flourishing in life. Journal of Health and Social Behavior.

[B14-ejihpe-15-00169] Keyes C. L. M. (2005). Mental illness and/or mental health? Investigating axioms of the complete state model of health. Journal of Consulting and Clinical Psychology.

[B15-ejihpe-15-00169] Keyes C. L. M., Bauer G. F., Hämmig O. (2014). Mental health as a complete state: How the salutogenic perspective completes the picture. Bridging occupational, organizational and public health: A transdisciplinary approach.

[B16-ejihpe-15-00169] Keyes C. L. M., Wissing M., Potgieter J. P., Temane M., Kruger A., van Rooy S. (2008). Evaluation of the mental health continuum-short form (MHC-SF) in Setswana-speaking South Africans. Clinical Psychology & Psychotherapy.

[B17-ejihpe-15-00169] Liu X., Wang J. (2024). Depression, anxiety and student satisfaction with university life among college students: A cross-lagged study. Humanities and Social Sciences Communications.

[B18-ejihpe-15-00169] Loureiro L. (2024). Acerca dessa coisa a que chamamos literacia em saúde mental. Revista De Enfermagem Referência.

[B20-ejihpe-15-00169] Loureiro L. M. J., Santos J., Loureiro C. (2025). Mental health continuum—Short form: Confirmatory factor analysis of competing models with adolescents from Portugal. European Journal of Investigation in Health, Psychology and Education.

[B19-ejihpe-15-00169] Loureiro L., Simões R., Rosa A. (2025). Depression: [Mental] Health literacy, stigma, and perceived barriers to help-seeking during transitions among undergraduate nursing students. Nursing Reports.

[B21-ejihpe-15-00169] Lovibond P. F., Lovibond S. H. (1995). The structure of negative emotional states: Comparison of the depression anxiety stress scales (DASS) with the beck depression and anxiety inventories. Behaviour Research and Therapy.

[B22-ejihpe-15-00169] Massano-Cardoso I. M., De S., Galhardo A. (2024). Ansiedade, depressão e stress em estudantes universitários deslocados da sua residência. Revista Portuguesa de Investigação Comportamental E Social.

[B23-ejihpe-15-00169] Matos A. P., André R. S., Cherpe S., Rodrigues D., Figueira C., Pinto A. M. (2010). Preliminary psychometric study of the Mental Health Continuum—Short Form—For youth, in a sample of Portuguese adolescents. Psychologica.

[B24-ejihpe-15-00169] Moeini M., Sarikhani-Khorrami E., Ghamarani A. (2019). The effects of self-compassion education on the self-efficacy of the clinical performance of nursing students. Iranian Journal of Nursing and Midwifery Research.

[B25-ejihpe-15-00169] Osafo A. B., Ohene-Darko S., Agormedah E. K., Omane-Adjekum C. (2025). Navigating the Transition to Higher Education: Understanding the Experience among First-Year University Students. Journal of Tertiary Education and Learning.

[B26-ejihpe-15-00169] Pais-Ribeiro J. L., Honrado A., Leal I. (2004). Contribuição para o estudo da adaptação portuguesa das escalas de Ansiedade, Depressão e Stress (EADS) de 21 itens de Lovibond e Lovibond. Psicologia, Saúde Doenças.

[B27-ejihpe-15-00169] Patelarou A., Mechili E. A., Galanis P., Zografakis-Sfakianakis M., Konstantinidis T., Saliaj A., Bucaj J., Alushi E., Carmona-Torres J. M., Cobo-Cuenca A. I., Laredo-Aguilera J. A., Patelarou E. (2021). Nursing students, mental health status during COVID-19 quarantine: Evidence from three European countries. Journal of Mental Health.

[B28-ejihpe-15-00169] Patterson C., Roberts M., Yousiph T., Robson G., Lewer K., Jay E.-K., Moxham L. (2025). Non-traditional mental health clinical placements: An effective means for reducing self-stigma in pre-registration nursing students. Journal of Psychiatric and Mental Health Nursing.

[B29-ejihpe-15-00169] Peris-Baquero Ó., Moreno-Pérez J. D., Navarro-Haro M. V., Díaz-García A., Osma J. (2023). Emotion dysregulation and neuroticism as moderators of group Unified Protocol effectiveness outcomes for treating emotional disorders. Journal of Affective Disorders.

[B30-ejihpe-15-00169] Pointon-Haas J., Waqar L., Upsher R., Foster J., Byrom N., Oates J. (2023). A systematic review of peer support interventions for student mental health and well-being in higher education. BJPsych Open.

[B31-ejihpe-15-00169] Purnama A., Susaldi S., Zahro Mukhlida H., Hasro Maulida H., Purwati N. H. (2021). Mental Health in Health Students during Coronavirus Disease-19: Systematic Review. Open Access Macedonian Journal of Medical Sciences.

[B32-ejihpe-15-00169] Rafati F., Nouhi E., Sabzevari S., Dehghan-Nayeri N. (2017). Coping strategies of nursing students for dealing with stress in clinical setting: A qualitative study. Electronic Physician.

[B33-ejihpe-15-00169] Shah N. S. M., Basri N. A., Ibrahim M. A., Hashim N. N. W. N. (2022). Correlation between emotion regulation and mental well-being among university students during COVID-19. Jurnal Psikologi Malaysia.

[B34-ejihpe-15-00169] Sonmez Y., Akdemir M., Meydanlioglu A., Aktekin M. R. (2023). Psychological distress, depression, and anxiety in nursing students: A longitudinal study. Healthcare.

[B35-ejihpe-15-00169] Tambling R. R., D’Aniello C. (2023). Mental health and relational health literacy among college students. American Journal of Health Promotion.

[B36-ejihpe-15-00169] Visier-Alfonso E. M., Sarabia-Cobo C., Cobo-Cuenca A. I., Nieto-López M., López-Honrubia R., Bartolomé-Gutiérrez R., Alconero-Camarero A. R., González-López J. R. (2024). Stress, mental health, and protective factors in nursing students: An observational study. Nurse Education Today.

[B37-ejihpe-15-00169] Westerhof G. J., Keyes C. L. M. (2009). Mental illness and mental health: The two continua model across the lifespan. Journal of Adult Development.

[B38-ejihpe-15-00169] Zhang J., Peng C., Chen C. (2024). Mental health and academic performance of college students: Knowledge in the field of mental health, self-control, and learning in college. Acta Psychologica.

